# Supplementary material for: Bacterial Profile and Antibiotic Resistance among Cancer Patients with Urinary Tract Infection in a National Tertiary Cancer Hospital of Nepal
Source: Trop Med Infect Dis. 2021 Apr 13;6(2):49. doi: 10.3390/tropicalmed6020049 (PMC8167765; doi:10.3390/tropicalmed6020049)
Supplement: Supplementary file 1 [file tropicalmed-06-00049-s001.pdf]

Supplementary Table S1. Results of antibiotic resistance testing on bacteria isolated from urine samples of cancer patients in B.P. Koirala Memorial Cancer Hospital, Bharatpur, Nepal between July 2018 to June 2019, stratified by cancer type

Cancer Type	Samples Cultured (n = 308)	Positive Cultures (n = 73)		Organisms Isolated in Positive Cultures (n)
		n	(%)	
Leukemia	105	11	(10.5)	*Citrobacter* (1), *E. coli* (6), *Enterococcus* (1), *Proteus* (1), *Pseudomonas* (1), *Staphylococcus* (1)
Urinary Bladder	33	15	(45.5)	*E. coli* (10), *Enterococci* (2), *Klebsiella* (1), *Staphylococcus* (2)
Lung	17	4	(23.5)	*E. coli* (3), *Staphylococcus* (1)
Colorectal	16	2	(12.5)	*Citrobacter* (1), *Klebsiella* (1)
Lymphoma	15	1	(6.7)	*Citrobacter* (1)
Ovary	14	7	(50.0)	*E. coli* (5), *Enterococcus* (1), *Klebsiella* (1)
Cervical	12	4	(33.3)	*E. coli* (1) *Pseudomonas* (2), *Staphylococcus* (1)
Oral cavity	11	1	(9.1)	*Klebsiella* (1)
Prostate	9	4	(44.4)	*Citrobacter* (1), *E. coli* (3)
Liver	6	0	(0.0)	
Stomach	6	2	(33.3)	*E. coli* (1), *Enterococcus* (1)
Brain and CNS	5	2	(40.0)	*E. coli* (1), *Staphylococcus* (1)
Breast	4	1	(25.0)	*Staphylococcus* (1)
Gall bladder	4	2	(50.0)	*Citrobacter* (1), *Klebsiella* (1)
Larynx	1	0	(0.0)	
Other*	43	14	(32.6)	*Citrobacter* (1), *E. coli* (10), *Enterococcus* (1), *Klebsiella* (1), *Staphylococcus* (1)
Not available	7	3	(42.9)	*E. coli* (2), *Klebsiella* (1)

CNS: central nervous system. *Other included cancers: bone, duodenum, endometrium, Ewing's sarcoma, Germ cell tumour, hemangioma, renal, pharynx, pancreas, penis, plasma cell, synovial sarcoma, testis, thymus, ureteric and Wilm's tumour.

Supplementary Table S2. Antibiotic resistance sensitivity testing results for organism isolates from urine samples of cancer patients in B.P. Koirala Memorial Cancer Hospital, Bharatpur, Nepal between July 2018 to June 2019 whose sample showed bacterial growth, stratified by AWaRe classification and antibiotic

Antibiotics, by AWaRe Category	*E. coli* (n = 42)			*Staphylococcus* (n = 8)			*Klebsiella* (n = 6)			*Citrobacter* (n = 6)			*Enterococcus* (n = 6)			*Pseudomonas* (n = 3)			*Proteus* (n = 1)		
	**S**	**R**	**NT**	**S**	**R**	**NT**	**S**	**R**	**NT**	**S**	**R**	**NT**	**S**	**R**	**NT**	**S**	**R**	**NT**	**S**	**R**	**NT**
**Access**																					
Nitrofurantoin	37	3	2	4	0	4	3	4	0	5	1	0	3	2	1	1	2	0	1	0	0
Ampicillin	0	28	14	2	4	2	0	5	2	0	5	1	2	2	2	1	2	0	0	1	0
Amikacin	18	13	11	4	3	1	3	2	2	2	1	3	1	3	2	1	0	2	0	0	1
Cotrimoxazole	5	22	15	2	2	4	1	4	2	0	4	2	2	0	4	0	0	3	0	1	0
Gentamycin	13	12	17	0	3	5	2	2	3	2	1	3	1	1	4	0	2	1	0	1	0
Cefalexin	0	20	22	2	4	2	0	3	4	0	4	2	0	0	6	0	0	3	0	0	1
Amoxicillin/Clavulanic acid	1	16	25	3	2	3	0	2	5	1	2	3	0	3	3	0	1	2	0	1	0
Ampicillin/Sulbactam	1	1	40	0	0	8	0	0	7	0	0	6	0	0	6	0	0	3	0	0	1
Doxycycline	0	0	42	0	0	8	1	0	6	0	0	6	0	0	6	0	0	3	0	0	1
**Watch**																					
Nalidixic acid	2	4	36	0	0	8	0	0	7	1	0	5	0	0	6	0	0	3	0	1	0
Ciprofloxacin	3	30	9	3	3	2	1	3	3	1	2	3	0	6	0	1	0	2	0	0	1
Norfloxacin	0	8	34	0	0	8	0	2	5	0	2	4	0	1	5	0	1	2	0	1	0
Ofloxacin	3	15	24	3	0	5	2	1	4	0	2	4	1	3	2	0	2	1	0	0	1
Levofloxacin	1	9	32	2	0	6	0	1	6	0	0	6	0	2	4	0	0	3	0	0	1
Ceftriaxone	5	19	18	1	2	5	1	4	2	0	2	4	1	3	2	1	1	1	0	0	1
Cefixime	1	23	18	0	3	5	0	2	5	0	2	4	0	1	5	0	3	0	0	0	1
Cefotaxime	0	8	34	1	2	5	0	2	5	0	2	4	0	1	5	0	0	3	0	0	1
Cefepime	2	19	21	1	2	5	0	2	5	1	2	3	1	1	4	1	0	2	0	0	1
Cefoperazone	0	1	41	0	0	8	0	0	7	1	1	4	0	0	6	0	0	3	0	0	1
Cefuroxime	0	6	36	0	4	4	0	0	7	0	0	6	0	0	6	0	0	3	0	0	1
Ceftazidime	1	1	40	0	0	8	0	1	6	0	0	6	0	0	6	0	1	2	0	1	0

Meropenem	0	1	41	0	0	8	0	0	7	0	0	6	0	0	6	0	0	3	0	0	1
Imipenem	7	1	34	0	0	8	1	0	6	0	0	6	0	0	6	0	0	3	0	0	1
Vancomycin	0	4	38	1	1	6	0	1	6	0	0	6	3	0	3	0	0	3	0	0	1
Piperacillin/Tazobactam	1	4	37	0	0	8	0	1	6	0	2	4	0	1	5	1	1	1	0	0	1
**Reserve**																					
Polymyxin B	5	2	35	0	0	8	2	0	5	0	0	6	0	1	5	3	0	0	0	0	1
Tigecycline	7	0	35	0	0	8	3	0	4	0	0	6	0	0	6	0	1	2	0	0	1

AWaRe: WHO Access, Watch, and Reserve classification of antibiotics, S: Sensitive, R: Resistant, NT: Not tested.
